# Mind-Body Therapies From Traditional Chinese Medicine: Evidence Map

**DOI:** 10.3389/fpubh.2021.659075

**Published:** 2021-12-10

**Authors:** Lissandra Zanovelo Fogaça, Caio Fabio Schlechta Portella, Ricardo Ghelman, Carmen Verônica Mendes Abdala, Mariana Cabral Schveitzer

**Affiliations:** ^1^Department of Preventive Medicine, Universidade Federal de São Paulo, UNIFESP, São Paulo, Brazil; ^2^Brazilian Academic Consortium for Integrative Health (CABSIn), São Paulo, Brazil; ^3^BIREME (Latin American and Caribbean Center on Health Sciences Information) - Pan American Health Organization/World Health Organization (PAHO/WHO), São Paulo, Brazil

**Keywords:** mind-body therapies, traditional Chinese medicine, Tai Chi, Qi Gong, evidence map, public health

## Abstract

**Background:** The mind-body therapies of traditional Chinese medicine include several intervention types and combine physical poses with conscious relaxation and breathing techniques. The purpose of this Evidence Map is to describe these different interventions and report related health outcomes.

**Methods:** This evidence map is based on the 3iE Evidence Gap Map methodology. We searched seven electronic databases (BVS, PUBMED, EMBASE, PEDro, ScienceDirect, Web of Sciences, and PschyInfo) from inception to November 2019 and included systematic reviews only. Systematic reviews were analyzed based on AMSTAR 2. We used Tableau to graphically display quality assessment, the number of reviews, outcomes, and effects.

**Results:** The map is based on 116 systematic reviews and 44 meta-analyses. Most of the reviews were published in the last 5 years. The most researched interventions were Tai Chi and Qi Gong. The reviews presented the following quality assessment: 80 high, 43 moderate, 23 low, and 14 critically low. Every 680 distinct outcome effect was classified: 421 as potential positive; 237 as positive; 21 as inconclusive/mixed; one potential negative and none no effect. Positive effects were related to chronic diseases; mental indicators and disorders; vitality, well-being, and quality of life. Potential positive effects were related to balance, mobility, Parkinson's disease, hypertension, joint pain, cognitive performance, and sleep quality. Inconclusive/mixed-effects justify further research, especially in the following areas: Acupressure as Shiatsu and Tuiná for nausea and vomiting; Tai Chi and Qi Gong for acute diseases, prevention of stroke, stroke risk factors, and schizophrenia.

**Conclusions:** The mind-body therapies from traditional Chinese medicine have been applied in different areas and this Evidence Map provides a visualization of valuable information for patients, professionals, and policymakers, to promote evidence-based complementary therapies.

## Highlights

- Mind-body therapies affects emotional, social, and health related outcomes.
- Evidence Map provides easy information for patients, professionals, and policy-makers.
- Positive effects include physiological indicators, mental health, and quality of life.
- Positive potential effects include metabolic indicators, pain, vitality, and well-being.

## Background

The WHO has been encouraging and strengthening the insertion, recognition, and use of traditional, complementary, and integrative medicines (TCIM), products, and their practitioners in national health systems at all levels of activity: Primary Care, Specialized Care, and Hospital Care, through the recommendations of the WHO Strategy on Traditional Medicine 2014-2023 (1).

Mind-body therapies (MBT) consider the interactions between brain, mind, body, and behavior and understand that emotional, mental, social, and spiritual factors can directly affect health (2). MBT includes Tai Chi, Qigong, Yoga, Meditation, and types of relaxation (e.g., breathing exercises, autogenic training, biofeedback, and neurofeedback) (3). Moreover, these therapies can be offered alone or together with conventional treatments, since self-efficacy by itself may produce physiological benefits (4).

Qi Gong covers several practices and it is important to describe their meaning. The “Qi” means the energy that gives rise to activities of human life and “Gong” concerns the regulation of Qi through practice. Qi Gong practices consist of two forms: Qi Gong dynamic (external) or Qi Gong meditative (internal). Qi Gong external involves movements of the whole body or limbs (e.g., Tai Chi and Baduanjin), while Qigong internal requires the maintenance of posture with subtle body movements when performing exercises involving breathing and the mind (e.g., Meditation and Mindfulness) (5).

Acupressure Shiatsu and Tuiná hold the same principles as Acupuncture, but are non-invasive and do not need sophisticated equipment, as they are technics that use pressure through the body itself, such as through fingers, at the Acupuncture meridians points, to activate the body's internal energy flow (Qi), contributing to the restoration of its internal balance (6). These practices are based on Traditional Chinese Medicine, with Tuiná (7) (Chinese massage therapy) being more developed in China, while Shiatsu is a form of Acupressure more developed in Japan, with reports since the 1920s by Tokujiro Namikoshi (8).

Since 2006, Mind-Body Therapies from Traditional Chinese Medicine (MBTTCM) are some of the 29 complementary therapies included in the Brazilian National Health System. MBTTCM are ancient and consist of skills used in mind-body exercises integrating controlled breathing, body posture, gentle, and synergistic movements with mind adjustments (9, 10).

Therefore, these practices may contribute to the psychological component of quality of life (11), self-care practices (12), hypertension, fall prevention, cognitive performance, osteoarthritis, depression, chronic obstructive pulmonary disease, pain, balance confidence, and muscle strength (13, 14). Because of the recent extension of the complementary therapies policy, the Brazilian Ministry of Health partner up with the Latin American and Caribbean Center on Health Sciences Information (BIREME - PAHO – WHO) and with the Brazilian Academic Consortium of Integrative Health (CABSIn) to develop complementary therapies evidence maps, including this one about Mind-Body Therapies from Traditional Chinese Medicine (MBTTCM). The objective of this Evidence Map is to describe these different interventions and report related health outcomes.

## Methods

The methodological steps of the evidence map are parallel to those involved in the initial stages of a systematic review. Although, systematic reviews look to collate a limited subset of the evidence base to answer a specific research question. However, the evidence map does not attempt to answer a specific research question instead guided by broader research objectives (15).

The Campbell Collaboration (16) suggests that any evidence and gap map are a systematic visual presentation of the availability of relevant evidence of effects for a policy domain. The map may be accompanied by a descriptive report to summarize the evidence for stakeholders such as researchers, research commissioners, policymakers, and practitioners.

This Evidence Map summarizes the interventions and health outcomes related to MBTTCM. These evidence maps considered six steps, each with a set of activities: (1) Search, (2) Selection, (3) Categorization, (4) Informetric, (5) Evidence map, and (6) Gaps (17). The method and results were reported according to Preferred Reporting Items for Systematic Reviews and Meta-Analyses (PRISMA) guidelines (18) and the International Initiative for Impact Evaluation (3iE) Evidence Gap Methodology (19). This Evidence Map was supported by a technical expert panel of librarians, practitioners, policymakers, and researcher content experts.

### Data Sources

Our search was conducted in several databases (BVS, PUBMED, EMBASE, PEDro, ScienceDirect, Web of Sciences, and PschyInfo), from each database inception to November 2019, looking for systematic reviews in English, Spanish, and Portuguese. The review question to guide the database search considered the following: (P) general population, (I) MBTTCM as intervention, (C) no comparator, and (O) health-related outcomes. We consulted topic experts and developed the search strategy together with Latin American and Caribbean Center on Health Sciences Information (BIREME), then entered the following expressions as shown in Table 1.

**Table 1:** Search strategy.

**Database**	**Search terms**
BVS	“Lian Gong” OR Taiji OR “Tai Ji” OR “Tai-ji” OR “Tai-Chi” OR “Tai-Chi-Chuan” OR “Tai Chi Quan” OR Taijiquan OR “IQi Gong” OR “Xiang Gong” OR “Dao Yin Bao” OR “Jian Gong” OR “Tai Chi Pai Lin” OR “Tai Ji Qi Gong” OR Qigong OR “Chi Kung” OR Kunye OR “Lien Chi” OR MJ: “tai ji” OR MJ: Qigong
PUBMED	Lian Gong [tw] OR Taiji [tw] OR Tai Ji [tw] OR Tai-ji [tw] OR Tai-Chi [tw] OR Tai-Chi-Chuan [tw] OR Tai Chi Quan [tw] OR Taijiquan [tw] OR IQi Gong [tw] OR Xiang Gong [tw] OR Dao Yin Bao [tw] OR Jian Gong [tw] OR Tai Chi Pai Lin [tw] OR Tai Ji Qi Gong [tw] OR Qigong [tw] OR Chi Kung [tw] OR kunze [tw] OR lin ch i [tw] OR tai ji [mh] OR Qigong [mh]
EMBASE	(‘lian gong':ti OR taiji:ti OR ‘tai ji':ti OR ‘tai-ji':ti OR ‘tai-chi':ti OR ‘tai-chi-chuan':ti OR ‘tai chi quan':ti OR taijiquan:ti OR ‘iqi gong':ti OR ‘xiang gong':ti OR ‘dao yin bao':ti OR ‘jian gong':ti OR ‘tai chi pai lin':ti OR ‘tai ji qi gong':ti OR qigong:ti OR ‘chi kung':ti OR kunye:ti OR ‘lien chi':ti OR ‘tai ji'/exp OR ‘qigong'/exp) AND [embase]/lim NOT ([embase]/lim AND [medline]/lim) AND (‘meta-analysis' OR ‘systematic review')
PEdro	Chinese Medicine, Tai Chi, Qi gong, Acupressure, Shiatsu
ScienceDirect	“Tai chi,” “acupressure”
Web of Sciences	“Tai chi”
PschyInfo	“Tai chi”

### Inclusion Criteria

Systematic reviews about MBTTCM interventions and adequate descriptions of health outcomes were eligible for inclusion. We defined systematic reviews studies that self-identified as such. All participants of all ages, regardless of health status, were eligible for inclusion in the review. We excluded systematic reviews that did not focus on MBTTCM health outcomes. We included interventions on Tai Chi, Qi Gong, Traditional Chinese Exercise, Baduanjin, Acupressure techniques Shiatsu, and Tuiná of any duration and follow up.

### Procedure

**Figure 1:**
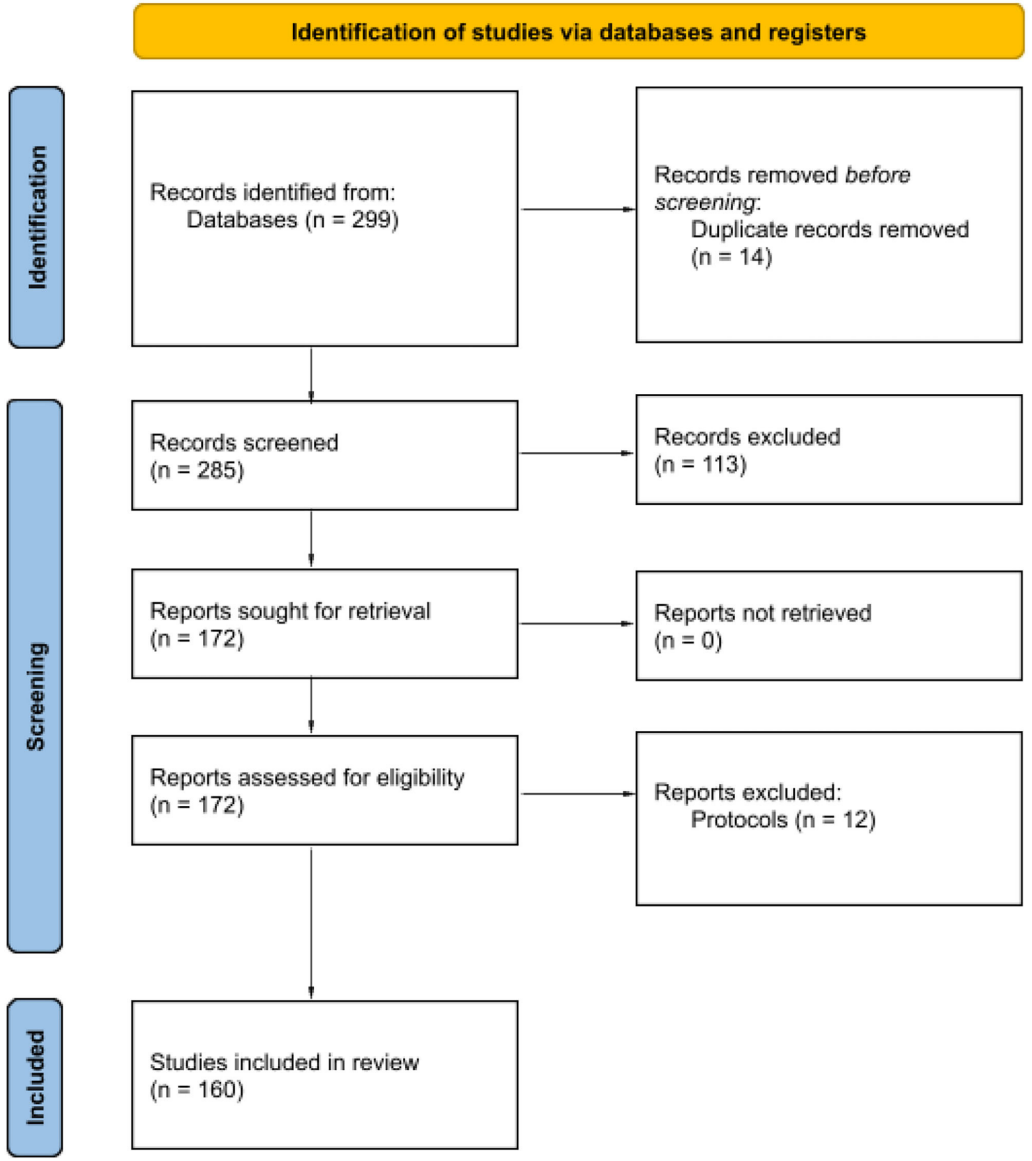
Flow diagram for systematic reviews included in the Mind-Body Therapies from traditional Chinese Medicine Evidence Map. Flow diagram template adopted from the PRISMA approach to systematic reviews (18).

Two blinded independent literature reviewers screened the systematic review search output through the Rayyan software. Citations deemed potentially relevant by at least one reviewer and unclear citations were obtained as full text. The full-text publications were screened against the specified inclusion criteria by two independent reviewers; disagreements were resolved through discussion. This process is displayed at the PRISMA Flow Diagram (18) (Figure 1).

We did not calculate the effect sizes in a meta-analysis nor did we provide the risk of bias assessments, but *Assessing the Methodological Quality of Systematic Review* (AMSTAR 2) was applied to analyze the quality (high, moderate, low and, critically low) of the included systematic reviews. The AMSTAR 2 by 16 item quality assessment analysis indicates confidence in the results of each review and describes the sources of bias: selection, measurement, and confounding (20). From each included systematic review, the intervention Tai Chi, Qi Gong, Baduanjin, Traditional Chinese Exercise, Shiatsu, and Tuiná was extracted, along with the main health outcomes (e.g., depression, hypertension, balance, physical function, mobility, risk of falls, well-being, pain) that were summarized across the included studies. The data about population, treatment effect (positive, potentially positive, mixed findings, potential negative and negative), estimates for health outcomes, and systematic review characteristics were retrieved.

### Data Synthesis

We developed a characterization matrix in Excel to synthesize the findings. This matrix included: Full-Text Citation; Interventions; Outcomes Group; Outcomes; Effect; Population (as described in each study); Database ID; Focus Country; Publication Country; Publication Year; Type of Review; Review Design; Study Design; Quality Assessment. The systematic review outcomes were drafted by one reviewer and discussed by the review team, and the matrix was discussed in two workshops organized by BIREME. We organized the Evidence Map considering the outcomes, effects, and quality assessment of the included systematic reviews. We use the interactive Tableau platform to graphically display all this information.

## Results

We identified 299 citations in the database search, 113 studies were excluded for not being systematic reviews, 172 were eligible for eligibility, 12 were excluded for being protocols, and 160 unique systematic reviews met the criteria for inclusion in the Evidence Map. Most of the reviews were published in 2017 and 2019. Tai Chi and Qi Gong were the most researched interventions.

**Figure 2:**
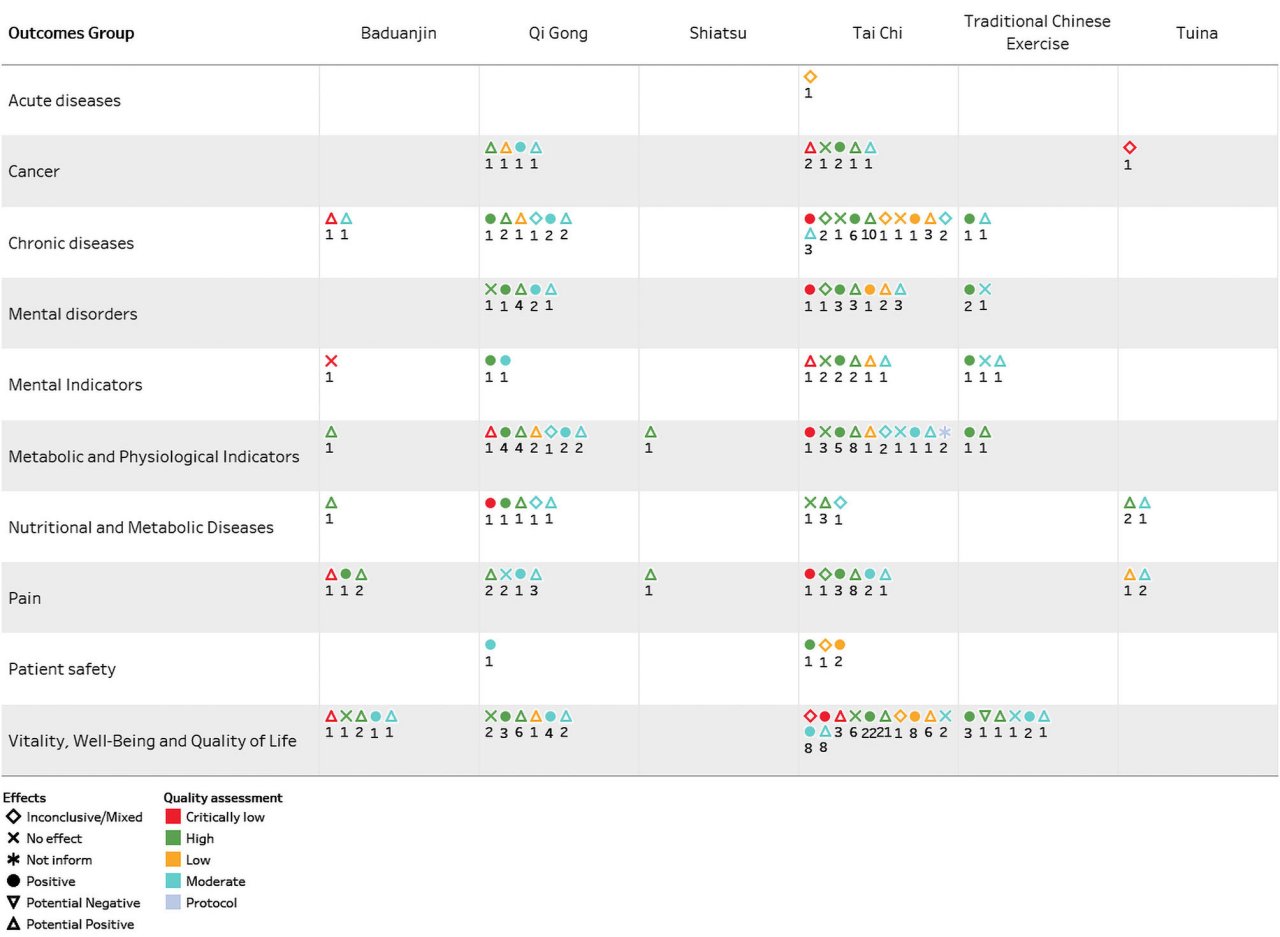
Outcome group, quality assessment, and effect by interventions of systematic reviews included in the Mind-Body Therapies from Traditional Chinese Medicine Evidence Map.

The results found in the 160 systematic reviews were divided into eight major outcomes groups: cancer; acute diseases; chronic diseases; physiological and metabolic indicators and nutritional and metabolic diseases; pain; patient safety; mental indicators and mental disorders; vitality, well-being, and quality of life. This evidence map spanned wide health outcomes, effects, and populations. The outcome group, quality assessment, and effect by interventions are presented in Figure 2.

### Interventions

The interventions were divided into two groups: Chinese bodily practices as Tai Chi, Qi Gong, Baduanjin, and Traditional Chinese Exercises; and manual stimulation of Acupuncture points (Acupressure) as Shiatsu and Tuiná. The Chinese bodily practices form was the most found in the reviews, highlighting the 313 Tai Chi and 107 Qi Gong interventions to distinct outcomes.

### Population

This evidence map analyzed data from the following populations: people with chronic disease (38), older adults (38), people in general (18), people with heart disease (16), people with cancer (12), women (11). and diabetics (8). In addition, smaller representations with adults (5), people with hypertension (3), patients with stroke (3), and people with osteopenia (2). Patients with schizophrenia, graduation students, children with autism, and women with cancer, each with only one study. Two reviews had mixed populations of adults, women, and older adults.

### Countries

The systematic reviews included analyzed data from the following countries: United States of America (50.3%), United Kingdom (23.7%), China (5.9%), Switzerland (5.1%), Singapore (2%), Germany (1.6%), Australia (1.4%), and England (1.25%).

### Effects and Outcomes

Mind-Body Therapies from Traditional Chinese Medicine (MBTTCM) was evaluated as an intervention for 109 distinct health outcomes. Every outcome effect was classified as 421 positives; 237 potential positives; 21 inconclusive/mixed; one potentially negative, and none no effect, several reviews had more than one effect. Chronic diseases highlight positive effects for rheumatoid arthritis, hypertension, diabetes mellitus, coronary diseases, osteopenia, and rates of glycemia and high-density lipoprotein (HDL). Mental disorders target depression, anxiety, cognitive performance, humor, well-being, and dementia. Vitality, well-being and, quality of life outcomes emphasize results as balance, physical function, mobility, exercise capacity, quality of life and, risk of falling.

**Figure 3:**
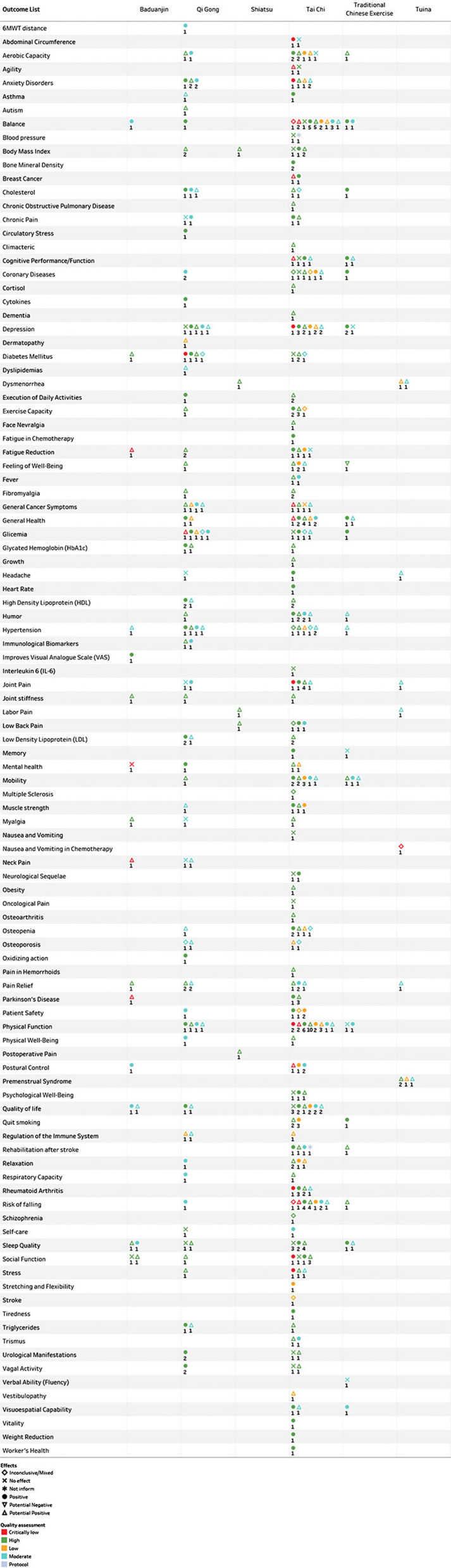
Outcomes, quality assessment, and effects by interventions of systematic interviews included in the Mind-Body Therapies from Traditional Chinese Medicine Evidence Map.

Figure 3 shows all these with more details as distinct ID counts divided by interventions vs. outcome list. Even as the colors show details about quality assessment and, the form shows details about the effects. Finally, the tags are labeled by distinct ID count and, the display is filtered on the effects of each intervention (Figure 3).

### Cancer

The cancer group results in the included systematic reviews were breast cancer, general cancer symptoms, cancer pain, fatigue, nausea, and vomiting. Among these, the MBTTCM showed positive effects for breast cancer, fatigue, and general cancer symptoms (21–23). The systematic reviews showed potential positive effects for general cancer symptoms (24–26), as well as no effects for cancer pain and adjuvant cancer treatment (27–30). One systematic review showed mixed effects for nausea and vomiting (31).

### Acute Diseases

The acute disease group results represent Tai Chi and Qi Gong interventions for stroke prevention. However, the effect of the single survey that related the results for stroke were inconclusive and mixed (32).

### Chronic Diseases

The chronic diseases group results were rheumatoid arthritis, coronary disease, Parkinson's disease, hypertension, diabetes, osteopenia, osteoporosis, fibromyalgia, osteoarthritis, chronic obstructive pulmonary disease (COPD), asthma, multiple sclerosis, and vestibulopathy. The effects of MBTTCM were positive for osteoarthritis (33–35), coronary diseases (36–39), hypertension (40, 41), cardiovascular disease and risk factors (32, 42), and diabetes (43, 44). The MBTTCM presented a potentially positive effect for Parkinson's disease (45–47), fibromyalgia (48, 49), osteoarthritis (50), premenstrual syndrome (51–53), COPD (27, 54–57), and vestibulopathies (58).

### Metabolic and Physiological Indicators and Nutritional and Metabolic Diseases

Mind-Body Therapies from Traditional Chinese Medicine (MBTTCM), as interventions for metabolic and physiological indicators and nutritional and metabolic diseases, showed positive effects for cholesterol, glycemia, and triglycerides (59, 60). Despite positive results, mixed-effects were also found in a study on cholesterol, glycemia, and no effect on blood pressure (36, 61–63).

### Pain

Mind-Body Therapies from Traditional Chinese Medicine (MBTTCM), as interventions for pain conditions, showed positive effects for headache, joint pain, chronic pain, and low back pain (64–67) Also, potentially positive effects were related to general pain, dysmenorrhea, neck pain, hemorrhoid pain, labor pain, post-operative pain, myalgia, and facial neuralgia (48, 49, 52, 68–74).

### Patient Safety

Mind-Body Therapies from Traditional Chinese Medicine (MBTTCM), as interventions—for patient safety, showed positive effects such as Tai Chi on the quality of life of patients with chronic disease, self-efficacy, psychological health conditions, and to prevent and manage cardiovascular disease (35, 75–79).

### Mental Indicators and Mental Disorders

In the mental indicators and mental disorders group, Mind-Body Therapies from Traditional Chinese Medicine (especially Tai Chi and Qi Gong) had positive effects for cognitive performance, memory, physical and psychological well-being, depression, anxiety, dementia, stress (56, 80–83), and mixed effect to schizophrenia (84).

### Vitality, Well-Being, and Quality of Life

Mind-Body Therapies from Traditional Chinese Medicine (MBTTCM) (especially Tai Chi and Qi Gong), as interventions in the vitality, well-being, and quality of life group, showed positive effects for balance, physical function, mobility (66, 82, 85–101), quality of life (102–104), and fall prevention (90, 97, 100, 105–107). There were also mixed effects for aerobic exercise, visuospatial capacity, fatigue-reducing, and cardiovascular disease (81, 108–110).

### Quality Assessment

The systematic reviews included were analyzed based on AMSTAR 2, resulting in the following quality assessment: 80 High, 43 Moderate, 23 Low, and 14 critically low. The systematic review's authors indicated some methodological flaws, highlighting population heterogeneity in the practice groups, period, and time of intervention.

### Limitations and Strengths

Even though Evidence Maps have several limitations, like the fact that we used only published reviews to provide an overview on the research on MBTTCM, more evidence, including qualitative studies, were not included. We did not calculate the effect sizes in a meta-analysis, nor provide the risk of bias assessments, but we tried to overcome these limitations by applying AMSTAR 2 to the quality assessment of the included systematic reviews.

In addition, the grouping of outcomes was review-content driven. Even though individual primary research studies would have more contributions to add to the analysis, this was not the focus of the Map. Besides, we were unable to avoid overlapping the included studies across reviews, but we did not repeat the results from updated reviews. We relied on the review author's skills in conducting systematic reviews, evaluation of primary studies quality, choice of outcomes, analysis of effects, susceptibility to publication, and outcome reporting bias.

Evidence maps are not designed to provide detailed and definitive information on the effectiveness of interventions. The implementation of the reviewed interventions in practice will require additional steps (e.g., identifying the optimal intervention format). Generally, evidence maps are a very broad overview of the evidence base, indicating areas in which research has been conducted, to help stakeholders interpret the state of the evidence to inform policy and clinical decision making.

Therefore, this evidence gap map can only provide a broad research overview, the findings showed more positive effects than potential negative or negative ones, including reviews of high, moderate, low, and critically low-quality assessments. The duration, period, and frequency of MBTTCM have not been analyzed and need more research.

The creation and publication of this evidence map consist in graphically representing the best evidence found, analyzed, and categorized, in addition to linking with the bibliographic records and full texts (when available) of the studies to facilitate access to information for all those interested.

### Research Gaps

The systematic reviews included did not clearly report the time of practices, frequency, more details of each practice, and duration of interventions. Therefore, the heterogeneity of the studies regarding the included participants, intervention characteristics, durations, and control groups may also limit the validity of the results. Furthermore, there have been no studies with pregnant women.

This Evidence Map will also not be able to answer more specific questions, such as the most appropriate method of applying Traditional Chinese Medicine Mind-Body Therapies, the difference between health services, adequate training of professionals, patient access, and self-application effects.

Future research, such as qualitative review surveys and evidence maps that only include systematic reviews of randomized clinical trials, are needed to answer refined questions, which are extremely important for the development of Traditional Chinese Medicine Mind-Body Therapies.

## Discussion

This evidence map for MBTTCM is based on 160 published systematic reviews and provides an available evidence broad overview of these interventions, related outcomes, and effects. It shows the volume of available research and highlights areas where the interventions showed positive effects.

The characteristics of the MBTTCM (e.g., Tai Chi, Qi Gong, Baduanjin, and Traditional Chinese Exercises) include low cost, moderate intensity, low technology, and low impact, and the possibility of practice by adults and older adults with chronic diseases (111). The movements are slow and rhythmic, linked together in a continuous sequence, and the body weight is shifted from one leg to another, challenging the balance control system to maintain its center within a changing support base (112).

Mind-Body Therapies from Traditional Chinese Medicine (MBTTCM) have been evaluated in different health conditions, including chronic diseases and mental disorders, and in vitality, well-being, and quality of life, assessed in a very broad population, from patients with chronic diseases to older adults.

This evidence map demonstrates that Tai Chi can significantly benefit adults and older adults with chronic diseases related to rheumatoid arthritis, hypertension, diabetes mellitus, coronary diseases, and osteopenia. Furthermore, significant improvements emphasize the results such as balance, physical function, mobility, exercise capacity, quality of life, and risk of falling. Highlighting beneficial effects for reductions in depression, disability falls, pain, and stiffness.

Tai Chi included significant improvements in cancers, chronic obstructive pulmonary disease, coronary heart disease, heart failure, hypertension, low back pain, osteoarthritis, osteoporosis, Parkinson's Disease, and stroke (113) as well as has favorable effects on depressive symptoms and quality of life of older adults (114), psychological well-being among persons with cardiovascular disease (115). Tai Chi can also improve strength, balance, balance confidence, mobility, gait, and executive function among older people (116, 117), reducing outcomes related to the extended frailty phenotype in older age adults (118).

The inconclusive/mixed-effects related to Tai Chi such as schizophrenia, aerobic exercise, visuospatial capacity, fatigue-reducing, and cardiovascular disease need more research.

The evidence map outcomes related Qi Gong to mental disorders scoped depression, anxiety, cognitive performance, mood, and feeling of well-being as well as chronic diseases such as diabetes, hypertension, metabolic syndrome, and cancer. Also, beneficial effects for reductions blood pressure, rates of glycemia, and HDL, pain, and improving risk factors metabolic syndrome were associated with Qi Gong.

Qi Gong may serve as a promising opportunity to improve psychological health domains such as the quality of life, depressive symptoms, fear of falling, and sleep quality in older adults (119), potentially having a beneficial effect on symptoms of anxiety (120), potentially effective to improve gait speed, balance, activities of daily living, and mobility to be a promising complementary therapy in Parkinson's Disease (121, 122), significant improvement in fatigue, and global distress in oncology patients (123).

Although this evidence map found positive effects of Qi Gong for diabetes, further research is suggested to debate these results. Also, Qi Gong applied to children with autism.

This evidence map involves 10 reviews of the Traditional Chinese Exercise (TCE) and only three reviews to present the Baduanjin results. The health outcomes for these interventions report positive and potentially positive effects related to chronic diseases such as diabetes, hypertension, coronary disease, osteoarthritis, and Parkinson's disease and the improvements emphasize results of fatigue, low back pain, neck pain, cognitive performance, psychological well-being and quality of sleep. The Baduanjin to osteoarthritis demonstrated a statistically significant improvement in pain, stiffness, and physical function (124), effective physical exercise intervention in patients with essential hypertension (125), is associated with statistically significant global cognitive function in patients post-stroke and the community of middle-aged and older adults. (126, 127). TCE can be potentially beneficial in alleviating cancer-related sleep disturbance (128), can effectively improve physical performance, balance, and muscle strength in the elderly population (129).

Although these studies include both high and moderate-quality assessments and could be considered for healthcare applications in these areas, further research with TCE and Baduanjin is needed.

Acupressure techniques such as Shiatsu and Tuiná are variants of Acupuncture and use hands and fingers to rub, knead or strike soft tissues and joints of the Acupuncture point regions in the body. They are non-invasive body practices and can be administered by the patients themselves (130).

This evidence map related to acupressure interventions showed potential positive effects involving dysmenorrhea, labor pain, nausea and vomit, low back pain, premenstrual syndrome, and pain relief. These effects appear in only seven reviews with high and moderate-quality assessment highlighting dysmenorrhea, nausea and vomit, and pain relief.

The Cochrane review demonstrates that acupressure probably has efficacy in reducing nausea and vomiting in women in labor, however, the stand-out evidence was generally low warranting further research (131). Acupressure may reduce pain intensity for pain management during labor (132, 133).

This Evidence Map demonstrated that acupressure could be considered for healthcare applications in these areas, nonetheless, further research is required.

Our map showed more positive effects and only one potential negative effect from a moderate quality review related to adverse effects and sense of well-being (134). The mixed effects justify further research and can help to guide different institutions' funding calls.

The outcomes and effects information of MBTTCM showed in these Maps will further advance our evidence-based knowledge of complementary therapies, such as that proposed by the complementary therapies policy in Brazil and promoted by the WHO MTCI 2014-2024 agenda.

## Conclusions

Mind-Body Therapies from Traditional Chinese Medicine (MBTTCM) have been applied in different areas and this map indicates 421 positive and promising health outcomes that need further research. Despite the outlined limitations, this evidence map provides a visualization of valuable information for patients, health practitioners, and policymakers, in order to promote evidence-based complementary therapies.

## Data Availability Statement

The original contributions presented in the study are included in the article/supplementary material, further inquiries can be directed to the corresponding author/s.

## Author Contributions

LF and MS drafted the manuscript. LF, MS, RG, CA, and CP designed the study and were involved in data acquisition and analysis. CA, MS, and LF designed and executed the search strategy. All authors were involved in the interpretation of the data, contributed to the final manuscript, read, and approved the final manuscript.

## Funding

The study is part of a project funded by the Brazilian Ministry of Health in partnership with the Latin American and Caribbean Center on Health Sciences Information (BIREME - PAHO - WHO) and the Brazilian Academic Consortium for Integrative Health (CABSIN) to develop complementary therapies Evidence Gap Maps, including Mind-Body Therapies of Traditional Chinese Medicine. The Brazilian Ministry of Health funded the project and BIREME-research partners conducted the study; collection; management; analysis; interpretation of the data; preparation, review, and decision to submit the manuscript for publication. The findings and conclusions in this publication are those of the authors who are responsible for its contents; the findings and conclusions do not necessarily represent the views of the Brazilian Ministry of Health and BIREME-PAHO-WHO.

## Conflict of Interest

The authors declare that the research was conducted in the absence of any commercial or financial relationships that could be construed as a potential conflict of interest.

## Publisher's Note

All claims expressed in this article are solely those of the authors and do not necessarily represent those of their affiliated organizations, or those of the publisher, the editors and the reviewers. Any product that may be evaluated in this article, or claim that may be made by its manufacturer, is not guaranteed or endorsed by the publisher.
